# A Ka-Band CMOS Transmit/Receive Amplifier with Embedded Switch for Time-Division Duplex Applications

**DOI:** 10.3390/mi16121309

**Published:** 2025-11-22

**Authors:** Peng Gu, Jiajun Zhang, Dixian Zhao

**Affiliations:** National Mobile Communications Research Laboratory, School of Information Science and Engineering, Southeast University, Nanjing 210096, China

**Keywords:** CMOS, low-noise amplifier, millimeter-wave 5G, power amplifier, transmit/ receive switch

## Abstract

Time-division duplex (TDD) transceivers have found broad utility in millimeter-wave 5G communication, radar and imaging applications. The co-design of the switch and transmit/receive (T/R) amplifiers becomes essential in optimizing the passive loss and chip size. This work presents a Ka-band T/R amplifier with an embedded switch topology. The amplification cores from the TX and RX channels reuse the matching network to the T/R common port, and the full combination of switching and matching structures is enabled within a compact two-winding transformer. Implemented in 40 nm CMOS technology, the proof-of-concept Ka-band T/R amplifier occupies a core chip area of 0.163 mm^2^. Experimental results show that it achieves a peak gain of 17.2 dB with a −3 dB bandwidth of 22.6–30.2 GHz in TX mode and a peak of 17.1 dB with a −3 dB bandwidth of 23.4–31.0 GHz in RX mode. The compact size and wideband gain response make the proposed T/R amplifier suitable for Ka-band TDD applications.

## 1. Introduction

Time-division duplex (TDD) transmission represents one of the most prevalent duplexing strategies, finding broad utility in applications such as millimeter-wave (mm-Wave) 5G communication networks, radar and imaging systems [1,2,3,4,5,6,7,8,9,10,11]. The realization of TDD operation in these systems requires the incorporation of transmit (TX) and receive (RX) modes together with the embedded mode switching capabilities. In contrast to standalone TX or RX designs [12,13,14,15,16], the integration of an additional switch element increases the design complexity of the TDD transceiver (TRX).

**Figure 1:**
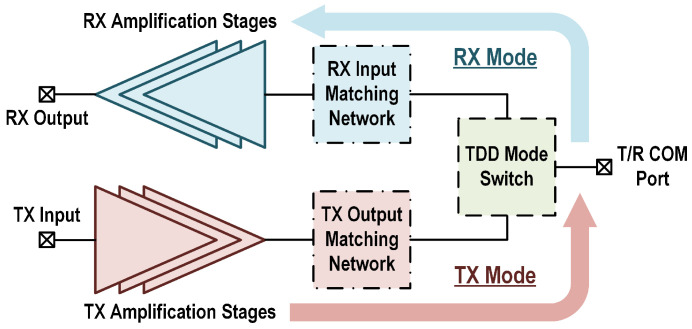
Full T/R amplifier topology for TDD applications, including the amplification stages, matching networks, and TDD mode switch.

The topology of a typical transmit/receive (T/R) front-end amplifier with an embedded mode switch for the TDD system is shown in Figure 1. It consists of the necessary amplification stages for the TX and RX chains, the TX output and RX input matching networks, and the TDD mode switch. Since the TX and RX chains generally target distinct performance requirements, separate implementations of the TX output and RX input matching networks allow the independent optimization of the two channels. The TDD mode switch contains three functional ports, which are connected to the RX input and TX output matching networks, and the T/R common port. This topology serves as a straightforward solution for TDD transceivers. However, implementing the matching networks and switch separately occupies a considerable chip area and increases the cost. Moreover, the inclusion of a TDD switch introduces additional losses in both the RX and TX chains, directly degrading the transceiver’s noise figure (NF) and output power, particularly at mm-Wave frequencies. These issues pose critical design challenges in minimizing the chip area and switch loss in TDD applications.

**Figure 2:**
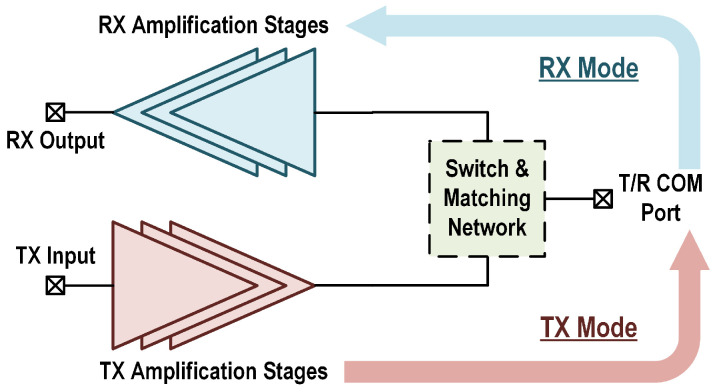
T/R amplifier topology with integrated hybrid network, simultaneously supporting impedance matching and TDD switching.

Hybrid networks that merge the switching operation into the impedance matching network, as depicted in Figure 2, are becoming a popular solution for TDD transceiver design. Partially or fully combining the matching networks with the T/R switch allows a reduced chip area and loss. In [5], the embedded switch transmission line topology is employed for the TDD operation of a 5G mm-Wave TRX front-end, which minimizes the TX loss with high branch impedance in the RX path. The RX side achieves a reduced chip area with the input matching network absorbed in the switch. In [17], a coupled-resonator-based T/R switch is proposed for the 5G mm-Wave GaN PA-LNA front-end module. The co-design and reuse of the switch and the matching networks allows a compact chip size. The TX output and RX input matching networks can further be integrated with the switching function. A three-winding transformer-based T/R switch enables concurrent RX and TX impedance matching while maintaining a compact chip footprint for bidirectional TRX designs [18,19]. The demand for higher transmit power drives the integration of multi-way PA in the TX channel, which in turn imposes additional challenges for switch design. In [20], the T/R switch is embedded into the two-way power combining network with MOS switches for mode configuration. In [21], a triple-coil-based load capacitance cancellation network is proposed to merge the dual PA paths and enable T/R switching within a compact transformer footprint. While these hybrid networks enhance the integration level of the T/R front-end, the partial combination of switching and matching still reduces the performance of either the TX or RX path, and full combination topologies with stacked coils usually require careful design to decouple the T/R modes.

This work proposes a Ka-band T/R amplifier with embedded switch operation. The TX side utilizes two capacitive neutralized common-source (CS) stages to achieve a sufficient gain and allow flexible bandwidth optimization. The RX side incorporates a capacitive gm-boosted common-gate (CG) stage to suppress noise and enhance the gain of the first stage. The RX gain and power are further improved with the second CS stage. The amplification cores of the TX and RX channels are combined and share the same passive matching network to the T/R common port. This allows the proposed T/R amplifier to adopt the full combination of switching and matching structures within a two-winding transformer. The proof-of-concept Ka-band T/R amplifier is implemented in 40 nm CMOS technology. Experimental results show that the T/R amplifier achieves a peak gain of 17.2 dB with a −3 dB bandwidth of 22.6–30.2 GHz in TX mode and a peak of 17.1 dB with a −3 dB bandwidth of 23.4–31.0 GHz in RX mode.

This article is organized as follows. Section 2 presents the architecture of the T/R amplifier. Section 3 details the circuit implementation. Experimental results are depicted in Section 4, followed by the discussion in Section 5 and the conclusions in Section 6.

## 2. System Architecture

**Figure 3:**
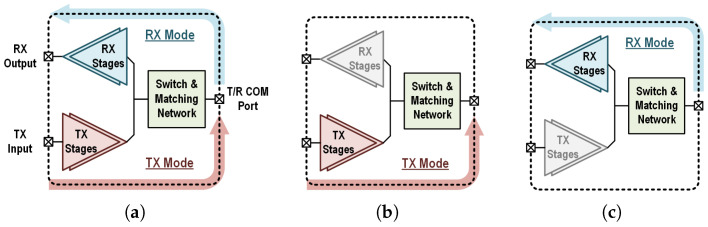
(**a**) Overall architecture of the proposed T/R amplifier. (**b**) Amplifier in TX mode with RX stages disabled. (**c**) Amplifier in RX mode with TX stages disabled.

The architecture of the T/R amplifier in this work is demonstrated in Figure 3a. The switch and matching network itself has two ports, which are connected to the external T/R common port and the internal TX and RX common nodes. The RX branch is turned off in TX mode and the TX branch is turned off in RX mode, as shown in Figure 3b,c. Fully combining the switching and matching functions, this topology represents a unique case of the hybrid network shown in Figure 2.

**Figure 4:**
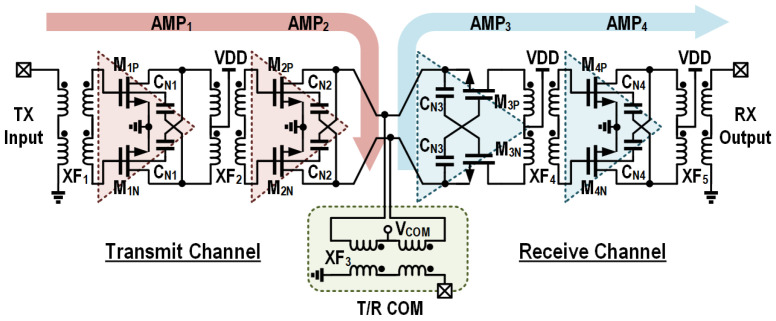
Schematic of the proposed T/R amplifier.

Figure 4 shows a detailed schematic of the proposed T/R amplifier. It consists of the TX and RX channels, each containing two amplification stages. The output side of the TX and the input side of the RX amplification cores are connected together and share the same matching network to the T/R common port. The common matching network is accomplished by a two-coil transformer XF_3_. The implementations of the TX and RX channels in the T/R amplifier are detailed in the following Section 3.

## 3. Circuit Implementation

### 3.1. Transmit Channel

**Figure 5:**
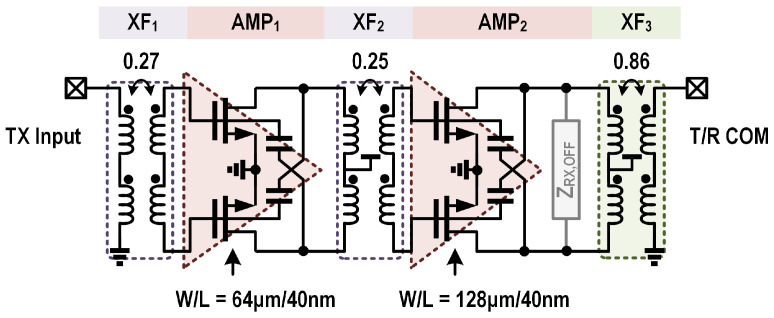
Schematic of the proposed T/R amplifier in TX mode.

The TX channel is composed of two capacitive neutralized CS amplification stages (i.e., AMP_1_ and AMP_2_) and three transformer-based matching networks (i.e., XF_1_, XF_2_ and XF_3_), as depicted in Figure 5. The power stage has a width of 128 μm to provide over 10 dBm output power with a reasonable margin, and the driver stage has a width of 64 μm for sufficient driving capabilities and improved efficiency of the TX channel. The adopted two-stage amplification topology ensures a reasonably high gain for the TX chain, which relaxes the driving capabilities required from the external TX stages.

**Figure 6:**
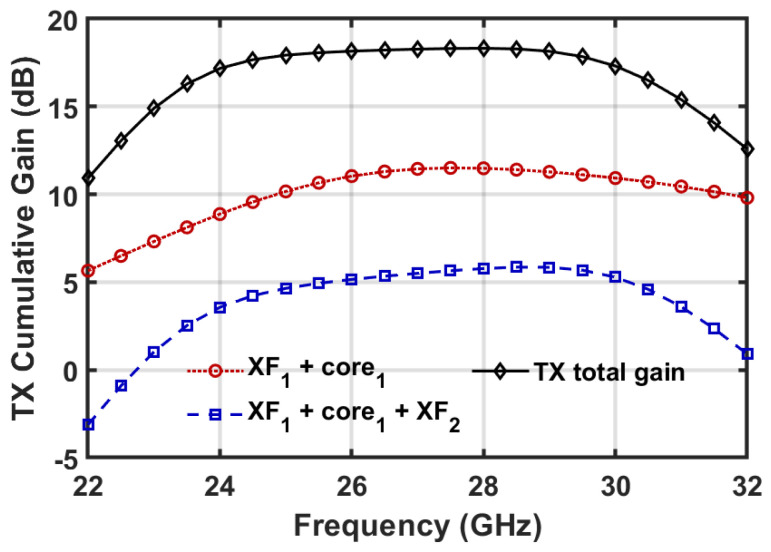
Simulated cumulative gain of the TX channel.

The collaborative operation of the three transformer-based impedance matching networks in the TX channel ensures a flat gain response across the target frequency band. In the TX channel, the XF_3_ transformer is first designed to transfer the optimal impedance—determined by jointly considering the TX power and RX noise simulations—to the external load (as detailed in Section 3.3), while the other two transformers are subsequently optimized to flatten the overall gain response. Figure 6 shows the simulated cumulative gain of the TX channel, which demonstrates the cooperative behavior of the matching transformers in achieving a flat gain response. Considering the high-Q resonating tank introduced by the input impedance of the CS amplification cores, the input and interstage transformers XF_1_ and XF_2_ have low coupling coefficients of 0.27 and 0.25, to achieve wideband impedance matching. In addition, XF_1_ and XF_2_ also serve to suppress the gain at the low-frequency side to compensate for the high-frequency gain roll-off introduced by the amplification cores. Ultimately, the TX channel achieves a flat overall gain across the 24–30 GHz frequency band, as shown in Figure 6.

### 3.2. Receive Channel

**Figure 7:**
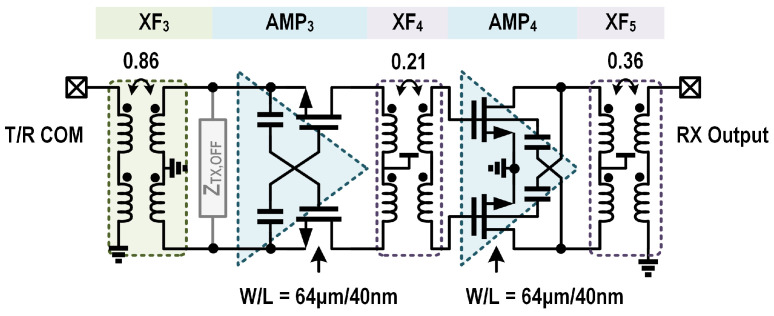
Schematic of the proposed T/R amplifier in RX mode.

The receive channel is composed of a capacitive gm-boosted CG stage and the following capacitive neutralized CS stage (i.e., AMP_3_ and AMP_4_), as shown in Figure 7. The pair of differential cross-coupled capacitors (C_*N*3_) for gm boosting has a value of 3 pF, which offers the source-to-gate inverting gain A ≈ −1 for the CG LNA stage, and it remains relatively compact regarding the practical layout of the amplification core. The width of the MOS devices in AMP_3_ is set to 64 μm, so that the RX noise and TX power matching impedances are close. The subsequent CS stage further enhances the gain of the RX chain to suppress the noise of external RX stages and improve the output power level.

**Figure 8:**
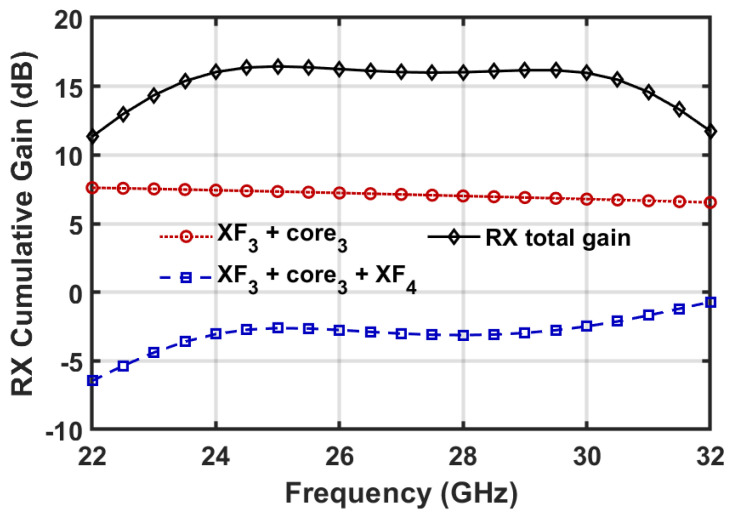
Simulated cumulative gain of the RX channel.

In RX mode, the flat gain response across the target frequency band is achieved by the collaborative operation of the three transformers (i.e., XF_3_, XF_4_ and XF_5_). The co-design of the TX power and RX noise determines the parameters for XF_3_, which transfers the external 50 Ω to the selected design point *Z*_*in*_, as detailed in Section 3.3. The transformers XF_4_ and XF_5_ are subsequently optimized to improve the overall gain flatness. In Figure 8, the simulated cumulative gain of the RX channel is demonstrated. Since the input impedance of the CG LNA stage constitutes a relatively low-Q resonating tank, the combination of XF_3_ and the CG amplification core exhibits low gain fluctuation, as depicted in Figure 8. While the second CS stage has high-Q input impedance, the interstage matching transformer XF_4_ has a low coupling efficient of 0.21 for wideband impedance matching. Meanwhile, XF_4_ also boosts the gain curve at the high-frequency side to compensate for the gain roll-off of the CS amplification core. Finally, XF_5_ accomplishes output impedance matching and contributes to a flat overall gain for the RX channel, as shown in Figure 8.

### 3.3. Embedded T/R Switch

As shown in Figure 4, the T/R switch and impedance matching functions are accomplished by the two-coil transformer XF_3_. In TX mode, the center tap of the primary coil in XF_3_ is switched to the VDD supply, which enables AMP_2_ in the TX channel and disables AMP_3_ in the RX channel. Meanwhile, XF_3_ serves as the output matching network for the TX channel. In RX mode, the center tap of the primary coil in XF_3_ is switched to the ground, which turns off AMP_2_ and turns on AMP_3_. Similarly, XF_3_ transforms the external standard 50 Ω to the matching impedance of the low-noise amplification stage.

**Figure 9:**
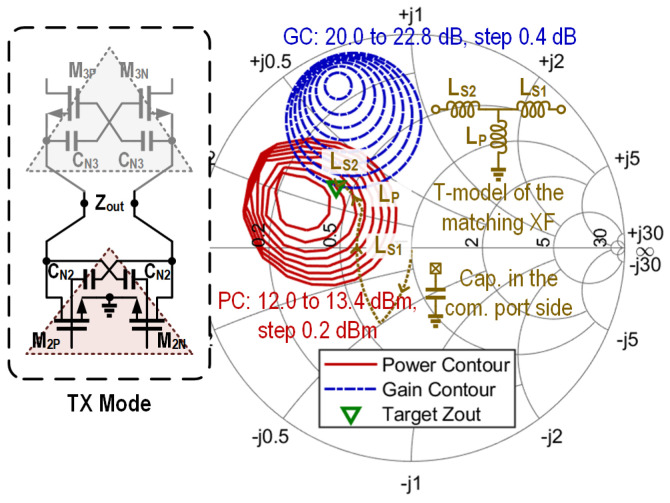
Output power and gain contours of the TX channel, together with the trajectory of the output matching network.

**Figure 10:**
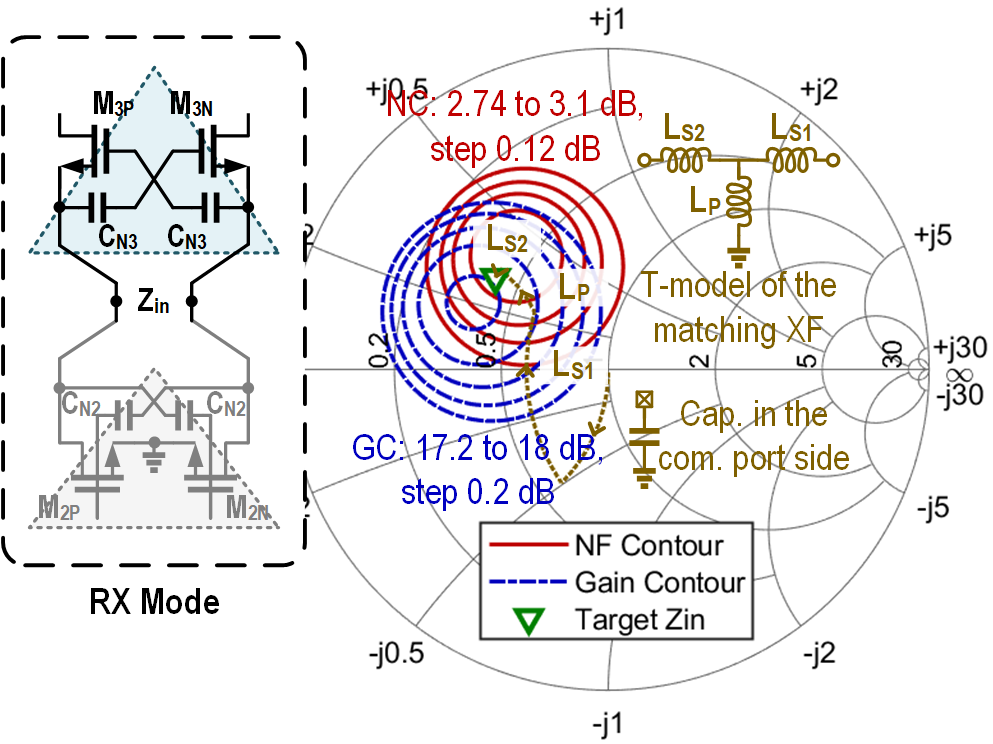
Noise figure and gain contours of the RX channel, together with the trajectory of the input matching network.

In TX mode, the RX path is turned off and presents relatively high input impedance to the connected node of the TX and RX amplification cores. The TX-to-RX leakage causes an approximately 0.4 dB decrease in output power. Similarly, in RX mode, the RX-to-TX leakage degrades the minimum NF by approximately 0.4 dB. In Figure 9, the simulated power contours and gain contours of the TX channel are depicted. Figure 10 shows the simulated noise figure contours and gain contours of the RX channel. Based on Figure 9 and Figure 10, the targeted impedances $Z_{out}$ and $Z_{in}$ for TX and RX are determined, which have an identical value of 20.8-j15. This allows good power matching at the TX side and ensures relatively low noise and a high gain on the RX side. The impedance matching trajectory is also illustrated in Figure 9 and Figure 10, which is achieved by the capacitor on the T/R common port side and the transformer XF_3_. The simulated insertion loss of XF_3_ is about 1.2 dB. Although the direct connection of AMP_2_ and AMP_3_ provides no isolation for the embedded switch, the overall T/R amplifier achieves over 31 dB simulated isolation between the TX input and RX output ports.

## 4. Experimental Results

**Figure 11:**
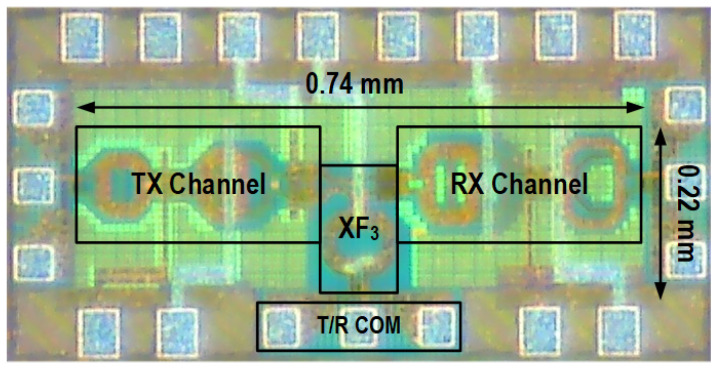
Micrograph of the proposed T/R amplifier.

The proof-of-concept T/R amplifier prototype is fabricated in 40 nm bulk CMOS technology and occupies a core area of 0.163 mm^2^, as shown in Figure 11. The performance of the chip is measured on a high-frequency probe station in both the TX and RX modes.

### 4.1. Transmit Mode

**Figure 12:**
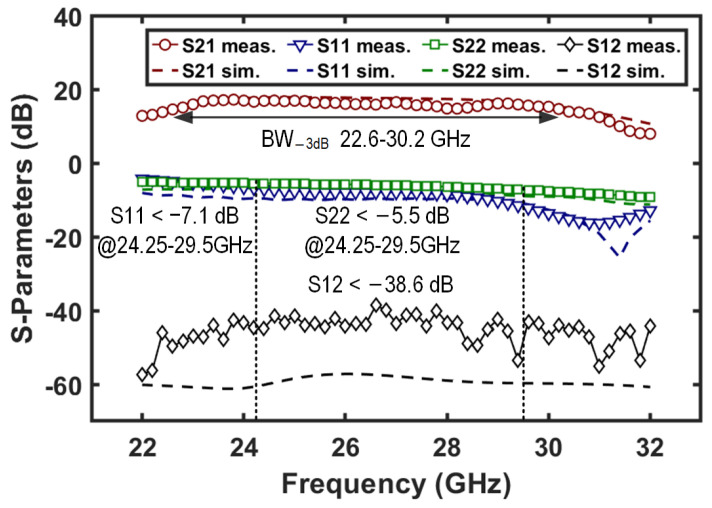
Measured and simulated S-parameters of the T/R amplifier in TX mode.

In TX mode, the two-stage amplifiers in the TX channel (i.e., AMP_1_ and AMP_2_) are enabled. The measured S-parameters of the chain are depicted in Figure 12. A peak gain of 17.2 dB is achieved with a −3 dB bandwidth of 22.6–30.2 GHz. The input and output return losses are better than 7.1 and 5.5 dB in the 24.25–29.5 GHz band. The reverse isolation remains above 38.6 dB across the measured frequency band.

**Figure 13:**
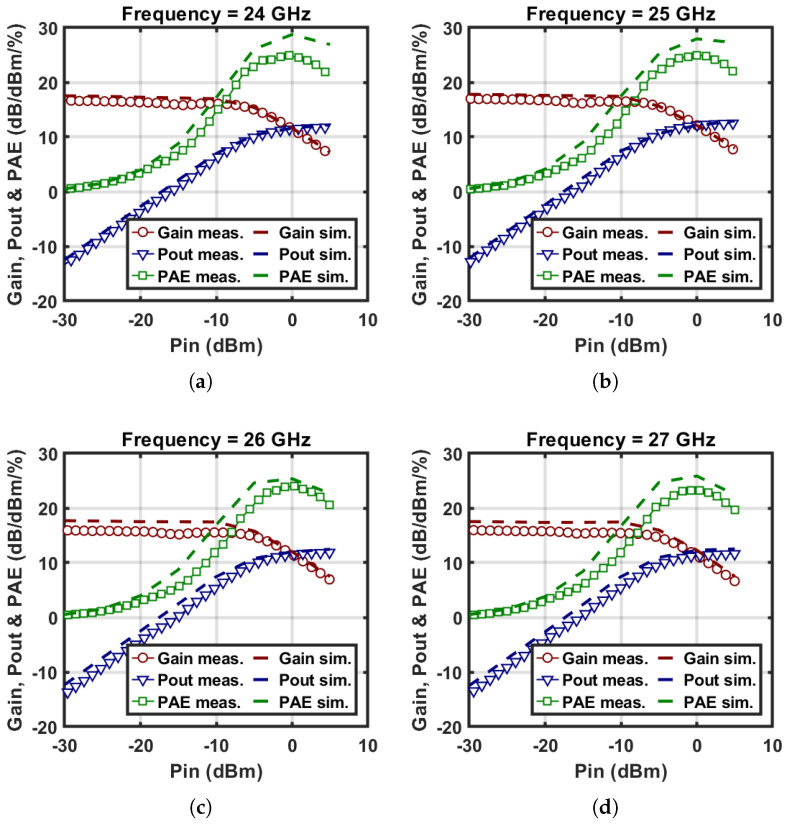
Measured and simulated power gain, output power, and PAE versus input power at the frequencies of (**a**) 24 GHz, (**b**) 25 GHz, (**c**) 26 GHz and (**d**) 27 GHz.

**Figure 14:**
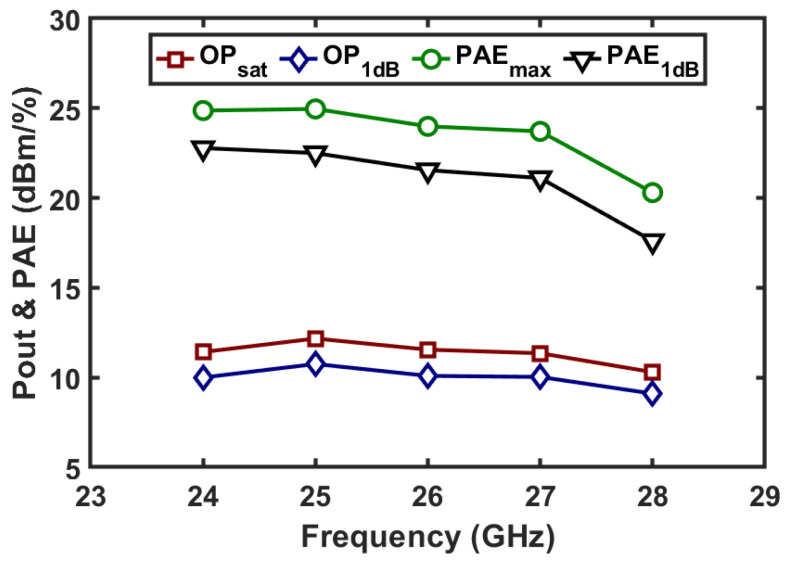
Measured output power and PAE versus operation frequency in TX mode.

The linearity of the TX channel is also evaluated with swept input power levels. The measured power gain, output power, and PAE of the two-stage amplifier in TX mode versus the input power are depicted in Figure 13. The amplifier achieves saturated output power of 11.4, 12.2, 11.6 and 11.4 dBm and a peak PAE of 24.9%, 25%, 24% and 23.2% at 24, 25, 26 and 27 GHz, respectively. In addition, at the 1 dB gain compression point, the amplifier provides over 10 dBm output power with a PAE value above 21.1% at 24, 25, 26 and 27 GHz. The output power and PAE versus frequency are depicted in Figure 14.

### 4.2. Receive Mode

**Figure 15:**
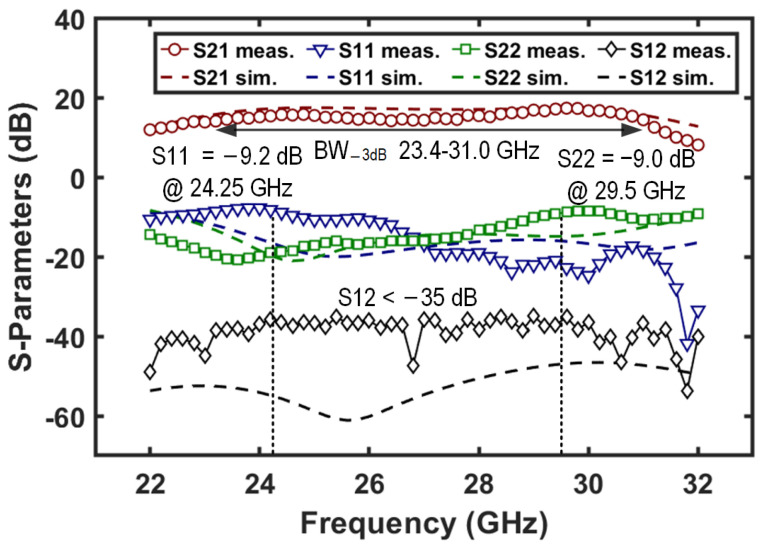
Measured and simulated S-parameters of the T/R amplifier in RX mode.

In RX mode, the two-stage amplifiers in the RX channel (i.e., AMP_3_ and AMP_4_) are enabled, which forms a low-noise receiving chain. The measured S-parameters of the chain are depicted in Figure 15. A peak gain of 17.1 dB is achieved with a −3 dB bandwidth spanning 23.4–31.0 GHz, indicating wideband gain performance. The input and output return losses are better than 9.2 and 9.0 dB in the 24.25–29.5 GHz band. Moreover, the reverse isolation with the two amplification stages remains above 35 dB across the measured frequency band.

**Figure 16:**
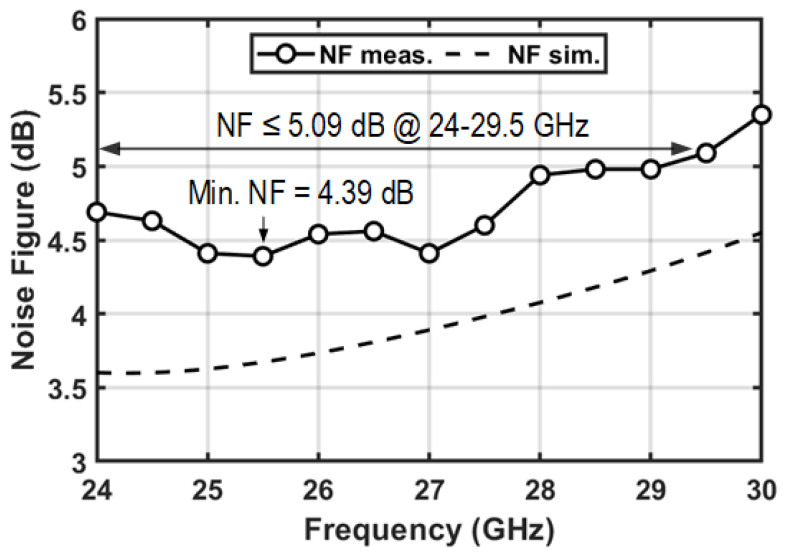
Measured and simulated noise figure of the T/R amplifier in RX mode.

In Figure 16, the measured NF of the RX channel is shown. The minimum NF of 4.39 dB is achieved at 25.5 GHz and the NF remains less than 5.09 dB across the 24–29.5 GHz band.

**Figure 17:**
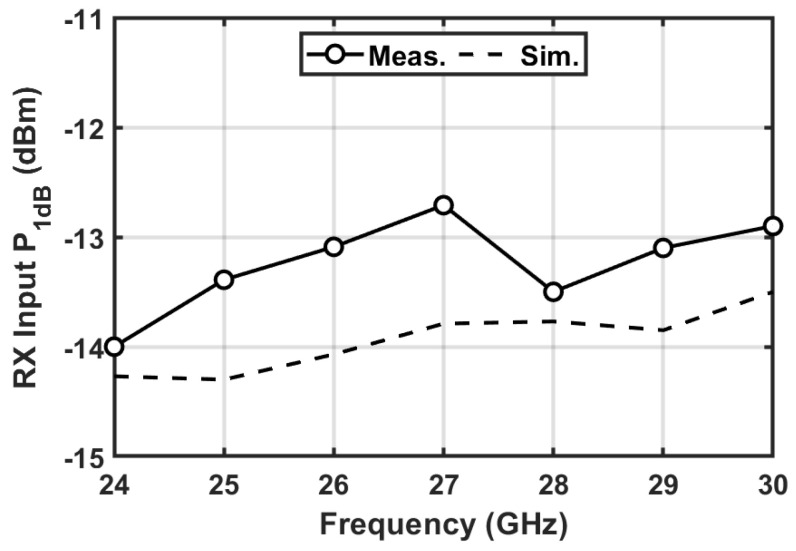
Measured and simulated input P_1dB_ of the T/R amplifier in RX mode.

In Figure 17, the measured input P_1dB_ of the RX channel is shown. The RX chain has a measured input P_1dB_ of −14 to −12.7 dBm across the 24–30 GHz band, which is relatively close to the simulated result.

## 5. Discussion

The performance of the proposed T/R amplifier is summarized and compared with the state of the art in Table 1. A compact two-winding transformer for hybrid switching and matching is introduced in this work, accompanied by capacitive gm boosting on the RX side. The proposed amplifier achieves wideband T/R operation in the Ka band, while ensuring a low NF, low DC power consumption, and a compact size simultaneously. The TX side has competitive efficiency among the designs in bulk CMOS. It should be noted that this work employs a single supply voltage of 0.9 V, avoiding the use of multiple voltage values or negative bias voltages (in GaN technology). While stacking FET techniques with a higher supply voltage and larger device width could be employed to improve the output power, this work targets a relatively low output power level of 10 dBm. Therefore, the single and low supply voltage, mitigating breakdown issues, makes it suitable for targeted applications; it supports moderate output power and sustains a compact chip size, a low NF, and low DC power consumption.

## 6. Conclusions

TDD applications require the co-design of switch, transmit and receive amplifiers for an optimal passive loss and chip size. A Ka-band T/R amplifier with an embedded switch topology is presented in this work, utilizing the full combination of switching and matching structures. The implemented Ka-band T/R amplifier in 40 nm CMOS occupies a core chip area of 0.163 mm^2^ and achieves a −3 dB bandwidth of 22.6–30.2 GHz in TX mode, as well as 23.4–31.0 GHz in RX mode. The compact size and wideband gain response make the proposed T/R amplifier suitable for Ka-band TDD applications.

## Figures and Tables

**Table 1:** Performance summary and comparison.

Reference	This Work	MWCL 2020 [22]	RFIC 2024 [23]	TMTT 2025 [17]	TMTT 2025 [18]	TMTT 2025 [19]	ISSCC 2025 [21]
Technology	40 nm CMOS	65 nm CMOS	28 nm CMOS	0.15 μm GaN-on-SiC	45 nm SOI CMOS	40 nm CMOS	65 nm CMOS
Topology	2-coil XF cap. gm-boost	CG-LNA reused sw	2-coil switch cap. XF	Coupled resonator sw	3-coil switch cap. XF	3-coil XF gate switching	Cap. cancel. in 2-way PA
Supply (V)	0.9	2.4	3/1	28/5/10	1	2.1/1.5	1.1/2.0/1.8
Frequency (GHz)	22.6–30.2 ^1^ (T) 23.4–31.0 ^1^ (R)	28 ^2^	27.3–35.4 ^1^ (T) 28.0–36.0 ^1^ (R)	24–30	19 (R) 29 (T) ^2^	60 ^2^	17.7–29.5
TX Gain (dB)	17.2	21	20.4	19.2	19.2	8.02	29.6
TX OPsat (dBm)	12.2	15.3	17.4	29.5	18.3	15.24	20.5
TX PAEmax (%)	25	25.1	17.2	17	28	9.35	21.8
RX Gain (dB)	17.1	19.6	17.3	22.8	16.2	11.29	25
RX NF (dB)	4.39–5.09	4.9 @28G	5.3–6.6	3.23–4.36	2.5 @19G	6.87 (sim.)	3.33–5
RX P_*DC*_ (mW)	17.3	28.8	63	425	36	78	48.7
Core Area (mm^2^)	0.16	0.22	0.1	5.46 ^3^	0.14	0.08	1.06

^1^ −3 dB bandwidth. ^2^ Center frequency. ^3^ Full chip area including pads.

## Data Availability

All data are included within the manuscript.
